# Extra-uterine Endometrial Stromal Sarcoma of the Stomach Mimicking a Gastrointestinal Stromal Tumour: A Case Report

**DOI:** 10.7759/cureus.105717

**Published:** 2026-03-23

**Authors:** Adam Mylonakis, Eleandros Kyros, Maria Karakeke, Eleni Karakeke, Andreas Panagakis, Pagona Kastanaki, Lysandros Karydakis, Alexandros Pergaris, Stratigoula Sakellariou, Alexandros Papalampros

**Affiliations:** 1 First Department of Surgery, Laiko General Hospital, National and Kapodistrian University of Athens, Athens, GRC; 2 First Department of Pathology, School of Medicine, National and Kapodistrian University of Athens, Athens, GRC

**Keywords:** endometrial stromal sarcoma, immunohistochemistry, letrozole, receptor, stomach neoplasms, tropomyosin-related kinase

## Abstract

Extra-uterine endometrial stromal sarcoma (EESS) is a rare mesenchymal tumour, and primary gastric involvement represents an exceptionally unusual presentation. Because such tumours present as ulcerated submucosal masses, they are easily misdiagnosed as gastrointestinal stromal tumours (GIST), making accurate, timely recognition critical for appropriate management.

A 53-year-old woman presented with a five-day history of epigastric pain, low-grade fever, and malaise. Computed tomography (CT) imaging revealed an 11 cm vascular mass on the greater curvature of the stomach with splenic-hilum and transverse-colon contact, without evidence of local invasion. Upper gastrointestinal endoscopy revealed a friable ulcerated lesion, and biopsies suggested a spindle-cell neoplasm favouring GIST. At laparotomy, the tumour was found to infiltrate the stomach, splenic hilum, colon serosa, and omentum. An en bloc partial gastrectomy, splenectomy, segmental colectomy, cholecystectomy, and omentectomy were performed; final pathology showed microscopic involvement of the gastric radial soft-tissue margin.

Histology demonstrated a primary gastric low-grade ESS with a vascular pattern and low mitotic index, while immunohistochemistry showed diffuse CD10/ER/PR positivity and absence of c-KIT, DOG-1, desmin, S100, SOX10, and STAT6, excluding GIST, leiomyosarcoma, PEComa, solitary fibrous tumour, and schwannoma. Targeted RNA-based sequencing identified a canonical JAZF1::SUZ12 fusion, confirming low-grade EESS. Given the tumour’s low-grade, hormone-receptor-positive biology and microscopic radial margin involvement, adjuvant endocrine therapy with letrozole (2.5 mg daily) was initiated. At the six-month follow-up, the patient remained asymptomatic with no radiological evidence of recurrence.

Primary gastric ESSS remains an exceptional finding and is easily mistaken for GIST. Accurate diagnosis and optimal management depend on comprehensive histology and immunohistochemistry, supported by fusion-gene testing, to guide individualised surgical and adjuvant management. Given the tumour’s potential for very late relapse, prolonged surveillance is warranted despite the favourable short-term outcome.

## Introduction

Endometrial stromal sarcomas (ESS) are a heterogeneous family of malignant stromal tumours that arise most often within the uterine corpus. In the current World Health Organization classification, ESS represents less than 1% of all uterine cancers and is the second-most frequent malignant mesenchymal tumour of the uterus. ESSs are divided into four entities: benign endometrial stromal nodules, low-grade ESS, high-grade ESS, and undifferentiated uterine sarcoma [1]. Of these, only the stromal nodule is considered benign, whereas the remaining categories are malignant.

Extra-uterine ESSs (EESSs) are exceptionally rare and are thought to originate from Müllerian stromal cells displaced by endometriosis or Müllerian rests [2,3]. The ovary, pelvic peritoneum, and bowel serosa are the most common ectopic sites, whereas primary gastric involvement remains exceptional, with only isolated cases reported [4,5]. Primary gastric ESS is diagnostically challenging because it may present as an ulcerated or submucosal spindle-cell lesion with clinical, endoscopic, and radiologic features closely overlapping those of gastrointestinal stromal tumour (GIST).

Current guidelines from the European Society of Gynaecological Oncology (ESGO), the European Reference Network for Rare Adult Solid Cancers (EURACAN), and the Gynecologic Cancer InterGroup (GCIG) advocate complete surgical excision as the cornerstone of treatment; adjuvant endocrine therapy is recommended for advanced or incompletely resected hormone-receptor-positive tumours [6].

We present a rare case of primary gastric EESS initially misinterpreted as a GIST, highlighting the diagnostic pitfalls of this entity, the value of immunohistochemical and molecular confirmation, and the implications for surgical and adjuvant management.

## Case presentation

A 53-year-old postmenopausal woman with a history of one caesarean delivery (25 years ago) presented to a tertiary referral centre in Athens, Greece, after five days of progressively worsening epigastric and diffuse abdominal pain accompanied by low-grade fever and malaise. She denied nausea, vomiting, gastrointestinal bleeding, weight loss, or changes in bowel habits, and she had no personal or family history of malignancy or chronic gastrointestinal disease.

On examination, she was afebrile, normotensive, and in no acute distress. The abdomen was soft, with mild-to-moderate tenderness in the epigastric and left-upper-quadrant regions but no rebound, guarding, or palpable masses; bowel sounds were normal.

Initial laboratory evaluation showed leukocytosis, thrombocytosis, mildly reduced haemoglobin, and an elevated C-reactive protein level. Liver biochemistry revealed mild elevations in aspartate aminotransferase, alanine aminotransferase, gamma-glutamyl transferase, and alkaline phosphatase. Coagulation parameters, bilirubin, renal function, and most tumour markers were within normal limits, apart from a mildly elevated CA-125. An overview of laboratory parameters is presented in Table 1.

**Table 1:** Laboratory results at presentation with corresponding normal reference values BUN: blood urea nitrogen; INR: international normalized ratio; aPTT: activated partial thromboplastin time; CA-125: cancer antigen 125; CEA: carcinoembryonic antigen; CA 19-9: cancer antigen 19-9; αLDH: lactate dehydrogenase Abnormal values are shown in bold.

Parameter	Result	Normal Reference Values
White blood cell count	12.75 × 10³ μL⁻¹	4.2–9.8 × 10³ μL⁻¹
Haemoglobin	11.6 g dL⁻¹	12.3–15.7 g dL⁻¹
Platelets	431 × 10³ μL⁻¹	160–390 × 10³ μL⁻¹
C-reactive protein	2.04 mg dL⁻¹	<0.5 mg dL⁻¹
AST	53 IU L⁻¹	8–42 IU L⁻¹
ALT	70 IU L⁻¹	7–49 IU L⁻¹
γ-Glutamyl transferase	113 IU L⁻¹	10–38 IU L⁻¹
Alkaline phosphatase	151 IU L⁻¹	40–130 IU L⁻¹
Total bilirubin	0.9 mg dL⁻¹	0.2–1.1 mg dL⁻¹
Direct bilirubin	0.2 mg dL⁻¹	0.0–0.3 mg dL⁻¹
Albumin	3.6 g dL⁻¹	3.8–5.2 g dL⁻¹
Creatinine	0.78 mg dL⁻¹	0.6–1.1 mg dL⁻¹
Urea (BUN)	16 mg dL⁻¹	7–22 mg dL⁻¹
INR	1.03	0.9–1.1
aPTT	29 s	25–34 s
CA-125	35.3 U mL⁻¹	<32 U mL⁻¹
CEA	2.1 ng mL⁻¹	<5.0 ng mL⁻¹
CA 19-9	12 U mL⁻¹	<37 U mL⁻¹
α-fetoprotein	2.8 ng mL⁻¹	<10 ng mL⁻¹
LDH	188 U L⁻¹	120–246 U L⁻¹

**Figure 1:**
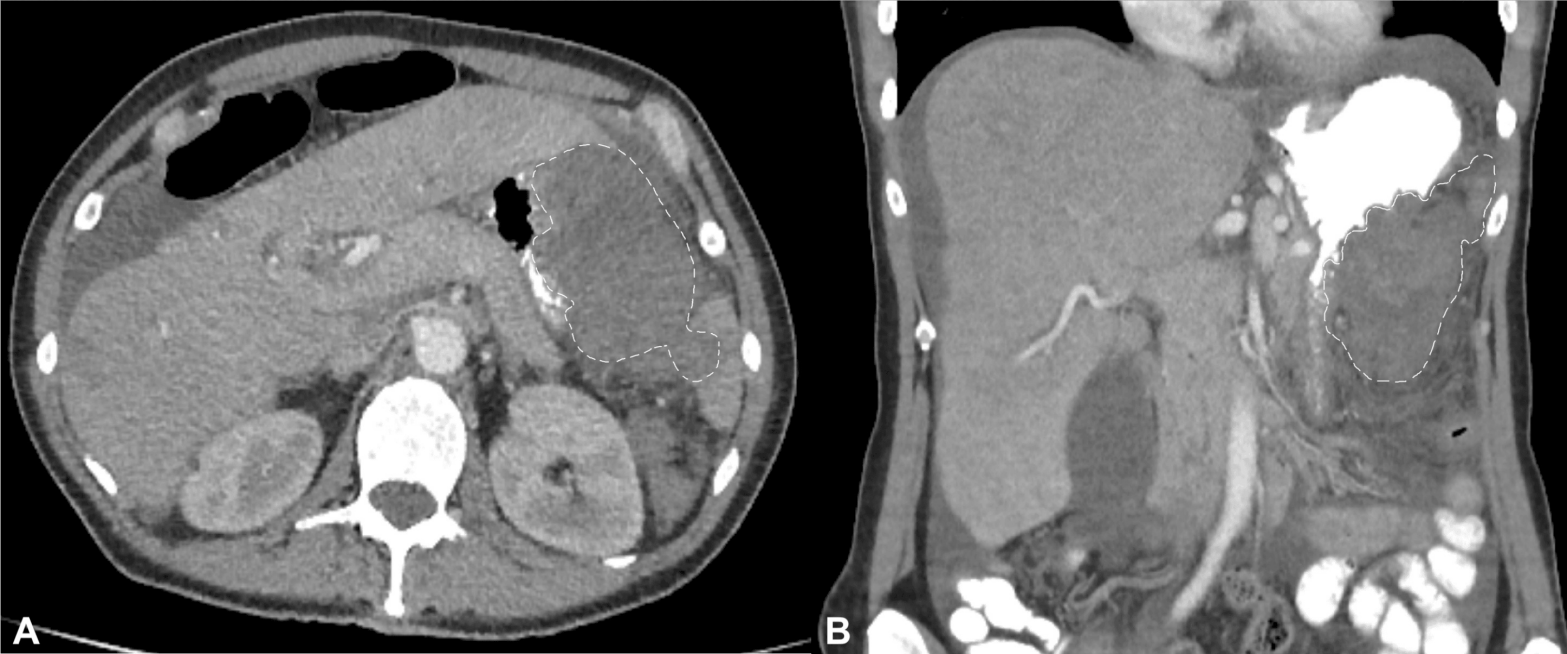
Contrast-enhanced CT of the abdomen (A) Axial and (B) coronal images demonstrate a well-circumscribed, heterogeneously enhancing soft-tissue mass (outlined) arising from the greater curvature of the stomach. The lesion measures approximately 11 cm, abuts the splenic hilum, and displaces the transverse colon inferiorly.

Contrast-enhanced CT of the abdomen and pelvis (Figure 1) demonstrated a well-vascularised, heterogeneous soft-tissue mass measuring roughly 11 cm along the greater curvature of the stomach. The lesion indented the gastric wall, displaced the transverse colon, and abutted the splenic hilum, with inflammatory stranding in the surrounding fat; however, there was no definite radiologic evidence of adjacent-organ invasion or distant metastatic disease.

Upper gastrointestinal endoscopy was subsequently performed and revealed an ulcerated, exophytic lesion on the greater curvature at the body-fundus junction. The mass was friable with an adherent clot and a firm base on probing. Multiple deep biopsies showed a spindle-cell proliferation, raising a strong suspicion of GIST. Immunohistochemical analysis was not performed on the endoscopic biopsy because the available tissue was limited and insufficient for a broad and reliable diagnostic panel. Taken together, the endoscopic appearance and biopsy findings supported a preoperative working diagnosis of GIST.

After a multidisciplinary review, an exploratory laparotomy was performed. Intraoperatively, a large tumour arising from the greater curvature, invading the splenic hilum and the subserosa of the transverse colon, was identified; a second nodule was noted in the greater omentum. Intraoperative ultrasound confirmed aneurysmal dilatation of the splenoportal confluence and showed no hepatic lesions. Given the tumour’s size, local extent, and involvement of adjacent structures, limited gastric resection was considered oncologically inappropriate. The patient, therefore, underwent en bloc partial gastrectomy, splenectomy, segmental transverse colectomy with primary side-to-side anastomosis, cholecystectomy, and omentectomy; intraoperative frozen section assessment was not performed. Cholecystectomy was performed to facilitate safe dissection in the hepatoduodenal region during this extensive upper abdominal multivisceral resection.

Postoperatively, the patient was extubated uneventfully and transferred to the intensive care unit and subsequently to the ward. On postoperative day 3, bilious drainage (~700 mL day⁻¹) appeared from a right subhepatic drain. MR-cholangiography localised the leak to the gallbladder bed, suggesting injury to an accessory duct of Luschka; conservative management with parenteral nutrition, a somatostatin analogue, and drain care led to spontaneous closure within one week.

On postoperative day 10, a follow-up CT scan obtained to evaluate for potential intra-abdominal collections showed no fluid collections but noted an acute thrombosis of the pre-existing portal-vein aneurysm; therapeutic low-molecular-weight heparin was started. She advanced to a soft diet, while drains and a central venous catheter were removed. The patient was discharged home on postoperative day 17, on full-dose anticoagulation.

**Figure 2:**
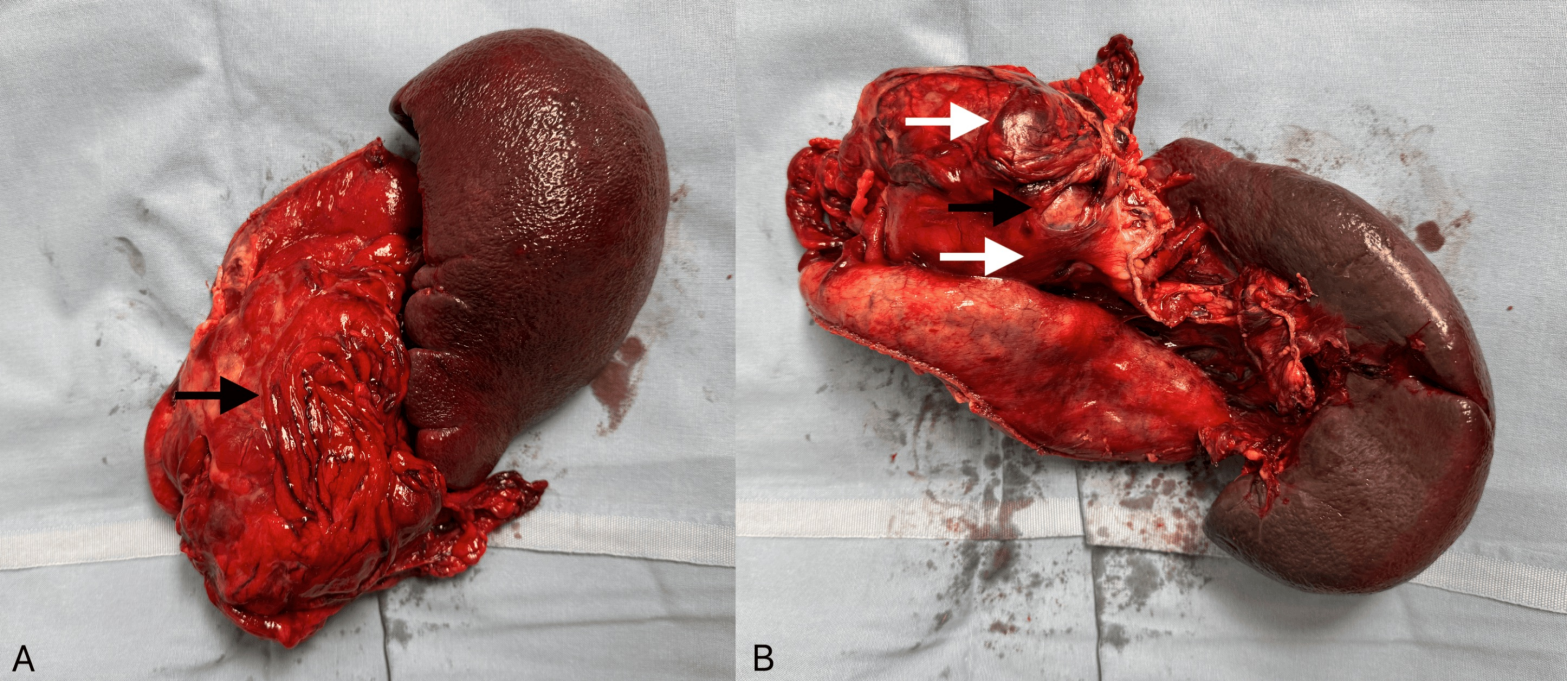
Anterior view (A) and posterior view (B) of a gross specimen of en bloc partial gastrectomy, splenectomy, and segmental transverse colectomy shows an exophytic, lobulated tumour along the greater curvature of the stomach with focal areas of haemorrhage (white arrows) and ulceration (black arrows). The lesion infiltrates perigastric fat and approaches the splenic hilum.

Pathologic examination of the surgical specimen revealed an exophytic tumour measuring 11 × 7.5 × 6 cm that ulcerated the gastric mucosa, penetrated the gastric wall, and spread into perigastric fat, the splenic hilum, and the serosal surface of the transverse colon; a 1.5-cm omental nodule showed identical morphology. Cut surfaces were fleshy with focal haemorrhage and necrosis (Figure 2).

**Figure 3:**
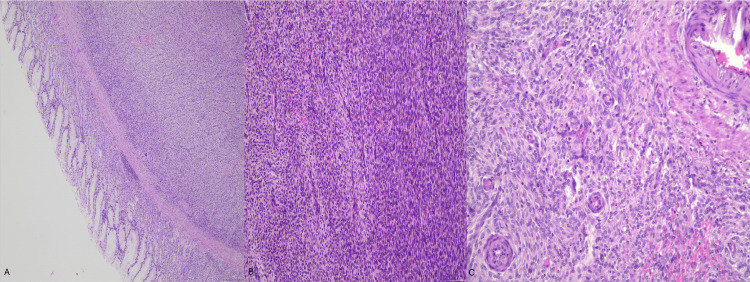
Histopathologic features of the gastric tumour on haematoxylin and eosin staining (A) Low-power view (×40) shows subserosal infiltration by the tumour. (B) Intermediate-power view (×100) shows a neoplasm composed of relatively uniform spindle cells with mild nuclear atypia and mild variation in cellularity. (C) High-power view (×200) shows characteristic perivascular whorling of tumour cells around small vessels, consistent with endometrial stromal differentiation.

Microscopically, all sites revealed a densely cellular proliferation of uniform spindle-to-oval cells possessing scant pale cytoplasm and finely stippled chromatin. The cells were disposed in intersecting fascicles and concentric cuffs around a delicate, branching capillary network, the classic “arborising” or “spiral arteriole” pattern of endometrial stromal differentiation. Tongue-like projections infiltrated adipose tissue and the colon subserosa, while hyaline fibrosis and haemorrhagic necrosis were scattered, but pleomorphism was minimal and atypical mitoses were absent; mitotic activity peaked at seven per 10 high-power fields (Figure 3). Six regional lymph nodes were negative for malignancy. The colonic mucosal and omental resection margins were free of tumour; however, microscopic tumour involvement was present at the gastric radial soft-tissue margin.

**Figure 4:**
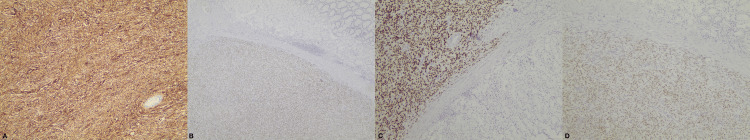
Immunohistochemical profile of the tumour (A) Diffuse and strong CD10 positivity in neoplastic cells (×100); (B) Diffuse nuclear positivity for WT1 (×40); (C) Diffuse nuclear positivity for progesterone receptor (×100); (D) Diffuse nuclear positivity for oestrogen receptor (×100).

Immunohistochemistry showed diffuse, strong nuclear oestrogen- and progesterone-receptor expression together with diffuse membranous-cytoplasmic CD10 positivity, confirming Müllerian stromal differentiation. Focal smooth-muscle-actin and weak CD99 positivity were present, whereas c-KIT, DOG-1, cyclin-D1, desmin, S100, SOX10, ALK, HMB-45, STAT6, and β-catenin were uniformly negative, effectively excluding GIST, leiomyosarcoma, PEComa, solitary fibrous tumour, and schwannoma (Figure 4). The Ki-67 proliferation index averaged approximately 30%, compatible with low-grade biology. Diffuse cytoplasmic pan-TRK staining initially suggested a possible NTRK rearrangement; however, targeted RNA-based next-generation sequencing identified a canonical JAZF1::SUZ12 gene fusion, confirming the diagnosis of low-grade ESS and excluding NTRK-driven disease.

Taken together, classic morphology, the canonical ER/PR/CD10 immunophenotype, low mitotic activity, absence of lineage-specific markers, and the presence of a JAZF1::SUZ12 fusion, these findings established the diagnosis of low-grade ESS involving the stomach, omentum, splenic hilum, and colon subserosa, pathologic stage pT4 pN0 (0/6). A comprehensive gynaecological evaluation, including pelvic MRI and transvaginal ultrasound, was subsequently performed and revealed no evidence of uterine or adnexal pathology, thereby confirming the extra-uterine origin of the tumour. Given the absence of uterine disease on imaging and history, the tumour was interpreted as a primary gastric low-grade ESS.

Given the tumour’s strong ER and PR expression, the patient’s postmenopausal status, microscopic margin involvement, and low-grade biology, the multidisciplinary team recommended adjuvant endocrine therapy with letrozole 2.5 mg once daily. At the six-month follow-up, she remained asymptomatic and radiologically free of disease recurrence.

## Discussion

ESS is a malignant Müllerian stromal tumour that accounts for less than 1% of all uterine cancers and roughly 10% to 15% of uterine sarcomas, corresponding to an incidence of ≈ 0.04 cases per 100,000 women-years [1,7]. Although most lesions arise in the uterine corpus, tumours can occur at extra-uterine sites, where they are referred to as EESS; such cases constitute less than 10% of all reported ESS [4]. While metastatic ESS to the stomach or other types of Müllerian stromal tumour arising from the stomach have been reported [8,9], primary gastric ESS is exceptionally rare, with two other cases reported in the literature [4,5]. This exceptional rarity contributes to the diagnostic difficulty of the lesion and explains why it is seldom suspected before definitive pathological assessment.

Primary gastric EESS usually presents with non-specific upper-abdominal symptoms rather than the uterine bleeding typical of intrauterine tumours. The two previously documented cases both involved middle-aged women with epigastric pain, dyspepsia, or upper gastrointestinal bleeding and were found endoscopically to harbour ulcerated submucosal masses on the greater curvature that mimicked GIST [5]. A broader review of EESS likewise shows abdominal pain, anaemia, and occult bleeding to be the most common clinical presentation when the gastrointestinal tract is involved [4]. Our patient fits this pattern: she arrived with a five-day history of worsening epigastric and diffuse abdominal pain, low-grade fever, and malaise, while endoscopy revealed an ulcerated, friable lesion on the greater curvature that was initially labelled as “probable GIST". These observations underline two practical points. First, gastric EESS produces vague, GIST-like symptoms and appearances, so a high index of suspicion is required for timely diagnosis. Second, full-thickness biopsy and immunohistochemistry are indispensable since clinical and endoscopic appearances can be highly misleading. This case underscores that primary gastric ESS, although exceptionally rare, should be considered in the differential diagnosis of ulcerated gastric spindle-cell lesions, particularly when the clinical and biopsy findings are suggestive but not definitive for GIST.

A spindle-cell mass in the stomach requires a detailed immunohistochemical work-up because several entities share an ulcerated submucosal appearance yet differ radically in biology and treatment. A GIST is the most common; virtually all gastric GISTs label for c-KIT (CD117) and DOG-1, markers that are absent in ESS, while true ESS shows diffuse CD10/ER/PR staining that GIST lacks [10,11]. Gastric leiomyosarcoma forms intersecting fascicles of eosinophilic spindle cells and is diffusely positive for desmin and smooth-muscle actin but negative for CD10 and hormone receptors [12,13]. Malignant PEComa may mimic ESS architecturally yet co-express melanocytic markers (HMB-45, Melan-A) and SMA, a profile not seen in ESS [14,15]. Solitary fibrous tumours can present as a serosal or submucosal plaque; their cells show nuclear STAT6 reactivity that reflects the NAB2-STAT6 fusion, whereas ESS is STAT6-negative and CD10-positive [16]. Gastric schwannoma exhibits peripheral lymphoid cuffs and diffuse S100/SOX10 expression with a complete lack of CD117 and DOG-1, distinguishing it from both GIST and ESS [17]. Rare entities also include inflammatory myofibroblastic tumour (IMT), which shows ALK-1 expression in about half of cases [18,19]; desmoid tumour with nuclear β-catenin positivity [20]; and metastatic or primary gastric melanoma, identified by S100, HMB-45, and melan-A staining [21]. In our case, the combination of classic morphology, diffuse CD10/ER/PR expression, WT1 positivity, absence of c-KIT and DOG-1, and lack of lineage-specific markers for smooth muscle, neural, melanocytic, ALK-rearranged, and STAT6-rearranged neoplasms effectively excluded the principal gastric spindle-cell mimics and supported the diagnosis of EESS.

Molecular analysis confirmed a canonical JAZF1::SUZ12 fusion, the most frequent genetic hallmark of low-grade ESS, detected in approximately 50%-60% of cases [2]. This rearrangement supports a low-grade, hormone-sensitive phenotype and clearly distinguishes the tumour from BCOR- or NTRK-rearranged high-grade ESS, which exhibit more aggressive behaviour. The identification of this fusion further supported the diagnosis of low-grade ESS and reinforced a low-grade, hormone-sensitive phenotype. In this clinicopathologic context, the molecular result argued against alternative high-grade molecular subtypes and supported endocrine-based postoperative management.

According to the 2024 ESGO/EURACAN/GCIG guidelines, complete surgical excision with negative margins is the cornerstone of treatment for both uterine and low-grade EESS [6]. Treatment should be undertaken in specialised centres to ensure the best patient outcomes [22]. Bilateral salpingo-oophorectomy is standard in uterine disease because ovarian oestrogen production may drive recurrence, but its value in extra-uterine primaries remains uncertain and should be individualised [6]. Our patient, therefore, underwent an en bloc partial gastrectomy, splenectomy, segmental colectomy, cholecystectomy, and omentectomy with the aim of achieving complete macroscopic clearance. Because the tumour showed strong ER and PR expression and the patient was postmenopausal, the multidisciplinary team recommended adjuvant endocrine therapy with letrozole 2.5 mg daily. The optimal duration of aromatase inhibitor therapy in extra-uterine low-grade ESS is not standardised and should be individualised according to menopausal status, completeness of resection, treatment tolerance, and recurrence risk. Durable disease control with aromatase inhibitors in residual or recurrent ESS, including extra-uterine cases, has been documented [23,24]. In the present case, the patient’s postmenopausal status reduced concern regarding ongoing ovarian oestrogen production and supported the use of letrozole without additional ovarian suppression surgery. TRK inhibitors (larotrectinib or entrectinib) were not indicated, as the tumour lacked NTRK rearrangement and exhibited canonical JAZF1::SUZ12-positive, low-grade biology. Conventional cytotoxic chemotherapy offers little benefit in low-grade, hormone-sensitive disease and is generally reserved for high-grade transformation or rapidly progressive, hormone-refractory tumours [25,26].

The postoperative bile leak from the gallbladder bed and thrombosis of the pre-existing portal venous aneurysm highlight the technical complexity of extensive upper abdominal multivisceral resection in this setting. These events also emphasise the importance of careful postoperative monitoring, appropriate cross-sectional imaging, and multidisciplinary management when uncommon tumours require non-standard resections.

Population-based analyses and large registry series confirm that low-grade ESS, whether uterine or extra-uterine, carries an excellent prognosis once an R0 resection is achieved: five-year disease-specific survival exceeds 90% for stage I-II tumours in the Surveillance, Epidemiology, and End Results (SEER) database and other cohorts [27,28,29], and remains around 80% to 90% at 10 years in contemporary EESS reviews [30]. Survival drops to around 50% in recurrent or stage III-IV disease, underscoring the importance of early diagnosis and complete cytoreduction [31]. Independent prognostic factors include surgical margin status, tumour stage, and the presence of high-grade transformation or mixed high-grade components, while extra-uterine location alone does not appear to confer a worse outcome when these variables are controlled [32-34]. In the present case, the locally advanced extent of disease, including involvement of adjacent structures and the omentum, together with microscopic radial soft-tissue margin involvement, indicates a higher risk of recurrence despite the low-grade histology. These features support the decision for adjuvant endocrine therapy and justify close long-term surveillance. Given the recognised potential for very late recurrence in low-grade ESS, follow-up should be prolonged, potentially lifelong, and include periodic clinical review with pelvic and systemic imaging according to recurrence risk and disease extent.

## Conclusions

Primary gastric EESS is exceedingly rare and may closely mimic GIST. Accurate diagnosis depends on careful morphologic evaluation supported by immunohistochemistry and molecular testing. Management requires individualised multidisciplinary planning, with complete macroscopic resection whenever feasible, consideration of endocrine therapy in hormone receptor-positive disease, and prolonged surveillance because of the risk of late recurrence.
